# Observed Immune Response Following Histotripsy in Metastatic Hepatocellular Carcinoma

**DOI:** 10.1155/crom/4245816

**Published:** 2026-04-17

**Authors:** Christopher Staley, Tahani Dakkak, Shane S. Robinson, Nelson A. Royall

**Affiliations:** ^1^ Department of General Surgery, Department of Graduate Medical Education, Northeast Georgia Medical Center, Gainesville, Georgia, USA; ^2^ Division of Research, Department of Graduate Medical Education, Northeast Georgia Medical Center, Gainesville, Georgia, USA; ^3^ General Surgery Residency Program, Department of Graduate Medical Education, Hepato-Pancreato-Biliary Surgery, Northeast Georgia Medical Center, Gainesville, Georgia, USA

**Keywords:** hepatocellular carcinoma, histotripsy, immunotherapy, liver cancer, observed immune response

## Abstract

**Background:**

Histotripsy is a novel, noninvasive, nonthermal tumor ablation therapy that uses focused ultrasound to mechanically disrupt tissue. Emerging evidence suggests that histotripsy may stimulate antitumor immune responses. To our knowledge, this is one of the first reported cases of histotripsy used in conjunction with systemic immunotherapy in a patient with hepatocellular carcinoma (HCC), resulting in an observed systemic immune response in nontarget lesions.

**Case Presentation:**

A 63‐year‐old male with noncirrhotic, nonviral multifocal metastatic HCC was treated with combination systemic immunotherapy (atezolizumab plus bevacizumab), followed by targeted histotripsy ablation of hepatic Segment II lesions. Given the presence of extrahepatic metastatic disease, liver transplantation was not a viable option. Posthistotripsy imaging demonstrated a partial response in the targeted lesion but partial responses in multiple nontarget lesions. This radiographic response occurred after discontinuation of systemic therapy due to toxicity. The patient maintained excellent functional status and continues to undergo histotripsy treatment.

**Conclusion:**

This case highlights the potential for histotripsy to elicit an abscopal‐like immune response in metastatic HCC when combined with immunotherapy. Histotripsy may serve as a valuable adjunct to systemic therapy, particularly in patients unable to tolerate prolonged immunotherapy. Further studies are warranted to explore this potential synergy in controlled clinical trials.

## 1. Introduction

Hepatocellular carcinoma (HCC) is the most common histologic subtype of primary liver cancer, accounting for approximately 90% of primary liver cancer cases [1, 2]. Despite advances in systemic and locoregional therapies, the 5‐year survival for HCC, in the United States, remains poor (15.6% overall and 21.5% disease‐specific) [2, 3]. Survival remains particularly limited in patients with multifocal or metastatic disease, even with aggressive locoregional therapies [4]. Recent developments in understanding the HCC tumor immune microenvironment have increased interest in immune‐mediated therapies. HCC is a type of neoplasm characterized by an immunosuppressive microenvironment that contributes to immune resistance through impaired antigen presentation, immunosuppressive activation and proliferation dysregulation, altered antitumor immune cell function, and overproduction of cytokines and chemokines [5]. Notably, a recent trial shared significant responses to the combination of both PD‐1 and CTLA‐4 inhibitors compared to PD‐1 monotherapy for locally advanced or metastatic HCC [6]. Ongoing studies are evaluating the role of combination locoregional therapies with systemic immunotherapies to enhance treatment efficacy [7]. Treatment is further complicated by cirrhosis, which limits locoregional therapies due to the risk of hepatic decompensation [8].

Intrinsic threshold histotripsy is a novel, nonthermal, and noninvasive, FDA‐approved modality for liver tumor ablation. Preclinical studies have demonstrated potential immune‐mediated responses within the tumor microenvironment through altered immune cell infiltration, cytokine/chemokine alterations, and altered tumor‐specific antigen recognition [9]. These findings suggest a potential role for histotripsy in eliciting systemic antitumor immune responses, including in nontarget lesions.

Given the novelty of the technique, clinical data regarding the use of histotripsy in metastatic HCC remains limited. Here, we present what is, to our knowledge, the first reported case of an observed systemic immune response in nontarget lesions following histotripsy in a patient with metastatic HCC.

## 2. Case Presentation

**Figure 1:**
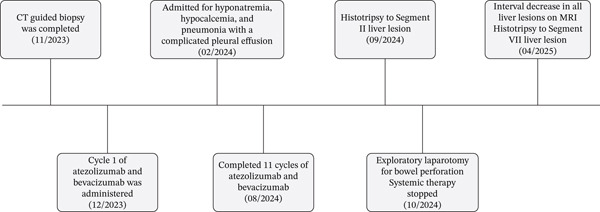
Case timeline of events.

We present the case of a 63‐year‐old male with a past medical history of coronary artery disease, hyperlipidemia, hypertension, hypothyroidism, obstructive sleep apnea, and traumatic brain injury. He presented to his primary care physician in November 2023 with complaints of significant fatigue, night sweats, unintentional weight loss, and a palpable mass in the right upper quadrant of the abdomen (Figure 1). He was functionally independent at baseline, with no history of significant tobacco use or alcohol abuse.

Initial computed tomography (CT) imaging of the chest, abdomen, and pelvis revealed hepatomegaly with multiple hepatic masses suspicious for malignancy, along with regional lymphadenopathy. Three dominant hepatic masses were visualized on CT imaging: a 14 × 11 cm mass in the right hepatic lobe and two lesions in the left hepatic lobe measuring 7 × 10 and 15 × 10 cm, respectively. Laboratory workup revealed an alpha‐fetoprotein (AFP) level of 41.2 ng/mL and a CA 19‐9 level of 12 U/mL (Figure 1).

CT‐guided core needle biopsy of a hepatic lesion revealed sporadic (noncirrhotic, nonviral), multifocal metastatic (N1+), moderately differentiated HCC. Molecular profiling demonstrated a PD‐L1‐negative tumor that was microsatellite stable with low tumor mutational burden and no actionable genetic mutations.

The patient was initiated on first‐line systemic therapy with atezolizumab plus bevacizumab in December 2023. After four cycles of combination therapy, his treatment course began to be complicated by multiple hospitalizations for treatment‐related adverse events, including hyponatremia, hypocalcemia, and pneumonia with complicated pleural effusion requiring thoracostomy tube placement in February 2024. Despite these complications, he completed 11 cycles of combination therapy, with interval imaging showing tumor size reduction (Figure 1).

Given his response to systemic therapy, he was evaluated for locoregional therapies including resection/ablation, arterial‐directed therapies, and radiation but was not a candidate due to tumor burden and his presenting comorbidities. He was also deemed ineligible for liver transplantation. In September 2024, he underwent intrinsic threshold histotripsy using the HistoSonics Edison system, targeting complete destruction of two 4.0 cm volumes to a dominant tumor within hepatic Segment II. He recovered from histotripsy without complications (Figure 1).

**Figure 2:**
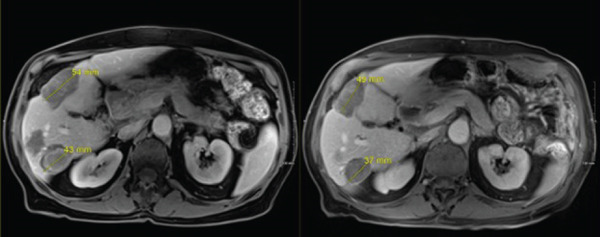
Two nontarget lesions with interval partial response.

**Figure 3:**
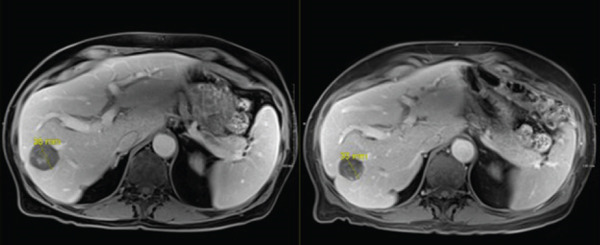
Additional nontarget lesion with partial response.

**Figure 4:**
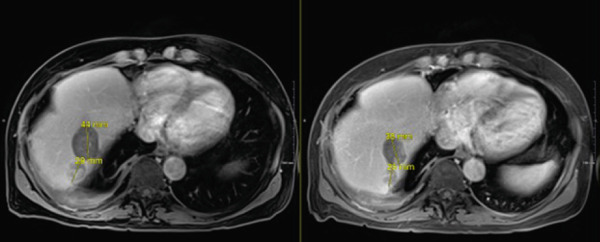
Target Sg II lesion with interval complete response (additional diffusion‐weighted imaging showing near complete resolution of diffusion enhancement).

**Figure 5:**
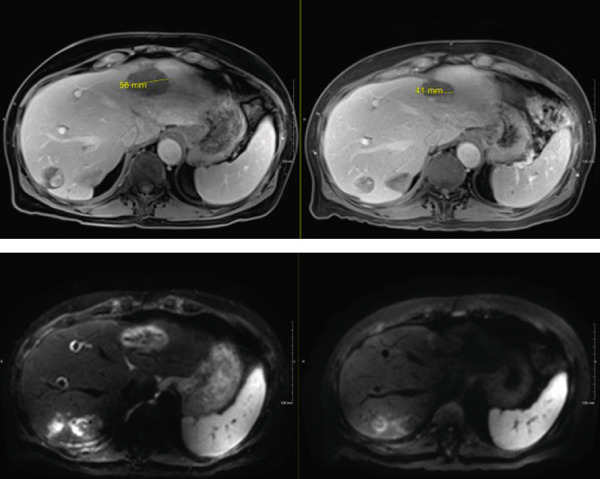
Additional nontarget lesions with partial response.

In late September 2024, he developed spontaneous small bowel perforation attributed to prolonged bevacizumab exposure and underwent emergent surgical resection. He similarly developed a Grade 2 hepatotoxicity requiring intermittent corticosteroid therapy. He recovered from these issues and maintained off systemic and locoregional therapies in the interval period. Surveillance MRI in April 2025 demonstrated partial responses in multiple nontarget lesions (Figures 2, 3, and 4) and a complete response in the targeted Segment II lesion (Figure 5). He subsequently elected to proceed with additional debulking histotripsy to the remaining liver lesions. At last follow‐up, the patient remains clinically stable with an Eastern Cooperative Oncology Group (ECOG) performance score of 0 and is doing well clinically. Written informed consent for publication was obtained prior to manuscript preparation.

## 3. Discussion

This case report presents a patient with metastatic, nonviral, noncirrhotic HCC who demonstrated radiographic partial responses in multiple nontarget hepatic lesions following debulking histotripsy of the liver in conjunction with systemic immunotherapy. To our knowledge, this represents one of the first clinical observations of a potential observed immune response (OIR) associated with histotripsy in HCC.

While the precise mechanisms underlying this effect remain under investigation, preclinical studies have proposed several hypotheses [10]. One primary mechanism involves the release of damage‐associated molecular patterns (DAMPs) during mechanical ablation. Unlike the thermal ablation technique, histotripsy avoids protein denaturation and preserves tumor antigens and DAMPs in an immunogenic form, which can be detected by circulating lymphocytes and presented by antigen‐presenting cells (APCs). Preservation of the surrounding vasculature and lymphatics may further facilitate immune cell infiltration and antigen access [10, 11]. As APCs can penetrate the tumor microenvironment, CD4+, cytotoxic CD8+ T cells, and dendritic cells are activated and can mount a response to both the targeted lesion and distant lesions [11].

The immune‐priming potential of histotripsy has been demonstrated in preclinical and early clinical studies. In the Phase I THERESA trial, a multicenter trial evaluating histotripsy in primary and metastatic liver tumors, two of seven patients (28.6%) demonstrated a reduction in untreated lesions, raising the possibility of histotripsy‐induced abscopal effects [11]. In murine models, Pepple et al. demonstrated that histotripsy promotes abscopal tumor growth inhibition through various mechanisms, including early release of high‐mobility group box Protein 1 (HMGB1), with increased transcriptional responses associated with innate immune activation and myeloid cell infiltration [12]. These findings align with our clinical observations and support further exploration of histotripsy as a tool to modulate tumor immunity. In this case, the patient had previously received a combination immune checkpoint blockade (atezolizumab plus bevacizumab), which had been discontinued due to adverse effects. The subsequent response of untreated lesions following histotripsy may suggest a residual immunological memory effect from prior therapy [13].

In conclusion, this case adds to the growing body of evidence demonstrating the potential impact that histotripsy has on improving immune response to nontarget liver lesions in the setting of HCC. When combined with systemic immunotherapy or used in patients who are unable to tolerate systemic agents, histotripsy may provide a synergistic therapeutic strategy. Clinical trials combining histotripsy with immune checkpoint inhibitors are warranted to better define its role in enhancing antitumor immunity in HCC.

**Nomenclature**
HCC: hepatocellular carcinoma
CT: computed tomography
AFP: alpha‐fetoprotein
Atezo/Bev: atezolizumab and bevacizumab
ECOG: Eastern Cooperative Oncology Group
DAMPs: damage‐associated molecular patterns
APCs: antigen‐presenting cells
HMGB1: high‐mobility group box Protein 1

## Author Contributions

All authors provided contributions to the draft and the development of this manuscript. C.S., T.D., and S.S.R. all contributed to the writing and development of the manuscript, and N.A.R. supervised and led the development of the manuscript as the senior author.

## Funding

No funding was received for this manuscript.

## Ethics Statement

Institutional Review Board (IRB) approval is waived as this is a case report.

## Consent

Patient consent has been obtained.

## Conflicts of Interest

The authors declare no conflicts of interest.

## Data Availability

The data that support the findings of this study are available on request from the corresponding author. The data are not publicly available due to privacy or ethical restrictions.
